# Molecular Characterization of Aquaporin 1 and Aquaporin 3 from the Gills of the African Lungfish, *Protopterus annectens*, and Changes in Their Branchial mRNA Expression Levels and Protein Abundance during Three Phases of Aestivation

**DOI:** 10.3389/fphys.2016.00532

**Published:** 2016-11-10

**Authors:** You R. Chng, Jasmine L. Y. Ong, Biyun Ching, Xiu L. Chen, Kum C. Hiong, Wai P. Wong, Shit F. Chew, Siew H. Lam, Yuen K. Ip

**Affiliations:** ^1^Department of Biological Sciences, National University of SingaporeSingapore, Singapore; ^2^Natural Sciences and Science Education, National Institute of Education, Nanyang Technological UniversitySingapore, Singapore; ^3^NUS Environmental Research Institute, National University of SingaporeSingapore, Singapore

**Keywords:** cocoon, desiccation, mucus, cell volume regulation, rehydration

## Abstract

African lungfishes can undergo long periods of aestivation on land during drought. During aestivation, lungfishes are confronted with desiccation and dehydration, and their gills become non-functional and covered with a thick layer of dried mucus. Aquaporins (Aqps) are a superfamily of integral membrane proteins which generally facilitate the permeation of water through plasma membranes. This study aimed to obtain the complete cDNA coding sequences of *aqp1* and *aqp3* from the gills of *Protopterus annectens*, and to determine their branchial mRNA and protein expression levels during the induction, maintenance and arousal phases of aestivation. Dendrogramic analyses of the deduced Aqp1 and Aqp3 amino acid sequences of *P. annectens* revealed their close relationships with those of *Latimeria chalumnae* and tetrapods. During the induction phase, there were significant decreases in the transcript levels of *aqp1* and *aqp3* in the gills of *P. annectens*, but the branchial Aqp1 and Aqp3 protein abundance remained unchanged. As changes in transcription might precede changes in translation, this could be regarded as an adaptive response to decrease the protein abundance of Aqp1 and Aqp3 in the subsequent maintenance phase of aestivation. As expected, the branchial transcript levels and protein abundance of *aqp1*/Aqp1 and *aqp3*/Aqp3 were significantly down-regulated during the maintenance phase, probably attributable to the shutdown of branchial functions and the cessation of volume regulation of branchial epithelial cells. Additionally, these changes could reduce the loss of water through branchial epithelial surfaces, supplementing the anti-desiccating property of the dried mucus. Upon arousal, it was essential for the lungfish to restore branchial functions. Indeed, the protein abundance of Aqp1 recovered partially, with complete recovery of mRNA expression level and protein abundance of Aqp3, in the gills of *P. annectens* after 3 days of arousal. These results provide insights into how *P. annectens* regulates branchial Aqp expression to cope with desiccation and rehydration during different phases of aestivation.

## Introduction

Lungfishes are an archaic group of freshwater fishes that belong to the class Sarcopterygii. They hold an important position in the evolution of vertebrates with regard to the water-land transition because they have lungs and some of them can survive emersion for an extended period. They share similarities with both fishes and amphibians, and are important species for studies on fish-tetrapod transition. Many neontologists consider lungfishes as a sister group of amphibians (Forey, 1986), while molecular phylogenetic studies favor the lungfishes as the closest living relatives of tetrapods (Takezaki et al., 2004; Hallström and Janke, 2009; Amemiya et al., 2013). However, paleontologists usually support a single origin of tetrapods from an extinct group (Rhipidistia) of lobe-finned bony fishes (Marshall and Schultze, 1992).

To date, there are six species of extant lungfishes worldwide, of which four (*Protopterus annectens, P. aethiopicus, P. amphibicus*, and *P. dolloi*) are found in Africa. African lungfishes are obligate air-breathers. In their natural habitats, African lungfishes can escape desiccation during the torrid season by burrowing into the mud and secreting mucus to form a cocoon in which it undergoes aestivation until the return of water. They can aestivate inside the subterranean mud cocoon for up to ~4 years during drought (see Ballantyne and Frick, 2010; Ip and Chew, 2010; Chew et al., 2015 for reviews). The mucus cocoon provides a physical barrier between the lungfish and the environment, and presumably protects the aestivating lungfish from evaporative water loss during the induction and maintenance phases of aestivation.

In the laboratory, African lungfishes can be induced to aestivate in completely dried mucus cocoon in plastic boxes (Chew et al., 2004; Ip et al., 2005; Loong et al., 2005), and there are three distinct phases of aestivation: induction, maintenance, and arousal. During the induction phase, the lungfish detects environmental cues and makes the required biochemical, physiological, structural, and behavioral changes for aestivation. It hyperventilates and secretes plenty of mucus mainly from the skin which turns into a cocoon after 6–8 days. The fish enters into the maintenance phase of aestivation when it is encased in a dried mucus cocoon, with absolutely no feeding and locomotor activities. In essence, the fish enters into a state of suspended animation, and many organs, including gills and kidneys, cease to function. For instance, the secondary lamellae are covered with mucus that pastes the lamellae together (Sturla et al., 2002), which remarkably reduce the vascular exchange area and render the gills non-functional during the maintenance phase of aestivation. With the addition of water, the lungfish can be aroused from aestivation; after arousal, it must rehydrate, excrete the accumulated waste products and make corrective changes to regain all physiological functions.

Aquaporins (AQPs) are a superfamily of integral membrane proteins that generally function for the selective passage of water or glycerol (Cerdà and Finn, 2010). To date, 17 AQP subfamilies (AQP0-16) have been described in a wide variety of animals (Finn et al., 2014), of which AQP0-12 have been categorized into three major groups: classical water-selective AQP (AQP0, −1, −2, −4, and −5), aquaglyceroporins (AQP3, −7, −9, and −10) permeable to glycerol, urea, and ammonia in addition to water, and unorthodox AQP (AQP6, −8, −11, and −12; Litman et al., 2009). Notably, Aqps are important water channels in the gills of many fishes and they may play a more crucial role in facilitating cell volume-regulatory fluxes of water than in transepithelial water transport (Madsen et al., 2015). It has been demonstrated that the mRNA expression level and protein abundance of *aqp3*/Aqp3 decrease significantly in the gills of seawater-acclimated fishes, including *Oreochromis mossambicus* (Watanabe et al., 2005), *Sparus sarba* (Deane and Woo, 2006), *Anguilla japonica* (Tse et al., 2006), *Dicentrarchus labrax* (Giffard-Mena et al., 2007, 2008), *Salmo salar* (Tipsmark et al., 2010), *Fundulus heteroclitus* (Jung et al., 2012), *Oryzias latipes* (Madsen et al., 2014), and *Oryzias dancena* (Kim et al., 2014). This may serve to reduce passive osmotic loss of water from the fish to the hyperosmotic environment (Madsen et al., 2015). By contrast, the branchial mRNA expression of *aqp1* is more varied among different fish species. In *S. salar*, the mRNA expression of *aqp1aa* is down-regulated in the gills upon seawater acclimation (Tipsmark et al., 2010). In the gills of *Acanthopagrus schlegeli* (An et al., 2008), *Takifugu obscurus* (Jeong et al., 2014), and *O. dancena* (Kim et al., 2014), the mRNA expression of *aqp1* are higher in fresh water than in seawater. However, there are no significant changes in the mRNA expression of *aqp1aa* in the gills of *Anabas testudineus* (Ip et al., 2013) and *D. labrax* (Giffard-Mena et al., 2007) upon seawater exposure. Apart from water transport, Aqp1/AQP1 can also facilitate CO_2_ or NH_3_ conductance (Chen et al., 2010; Geyer et al., 2013; Kaldenhoff et al., 2014), while Aqp3/AQP3 can also support NH_3_ or urea fluxes (Litman et al., 2009; Li and Wang, 2014).

Konno et al. (2010) have cloned and sequenced *aqp0* and *aqp0p* from the kidney, lung, and eyes of *P. annectens*, and reported the presence of the vasopressin-vasotonic receptor-Aqp0p axis, which was functional only during aestivation, in the kidney. They have also submitted the sequences of *aqp1* and *aqp3* to the NCBI database (Accession: AB499798.1 and AB499799.1, respectively) and incorporated them into the phylogenetic analysis of *aqp0* and *aqp0p* (in Figure 2 of Konno et al., 2010). However, no work has been conducted on any *aqp*/Aqp isoforms in the gills of *P. annectens*, and no information is available on how branchial *aqp*/Aqp expression would respond during the three phases of aestivation. Therefore, this study was undertaken to obtain the complete coding cDNA sequences of *aqp1* and *aqp3* from the gills of *P. annectens*, and to elucidate whether there were gill-specific *aqp1* and *aqp3* isoforms. It was hoped that the deduced Aqp1 and Aqp3 amino acid sequences would shed light on the phylogenetic relationship between *P. annectens* and other animals. Furthermore, efforts were made to determine by quantitative real-time PCR (qPCR) the mRNA expression of *aqp1* and *aqp3* in the gills of *P. annectens* kept in fresh water (control), or undergoing 6 days (the induction phase) or 6 months (the maintenance phase) of aestivation, or after 1 day or 3 days of recovery from 6 months of aestivation (the arousal phase). Based on the deduced Aqp1 and Aqp3 sequences, custom-made anti-Aqp1 and anti-Aqp3 antibodies were developed for the determination of their protein abundance in gills through Western blotting. The hypotheses tested were that aestivation would induce changes in the mRNA and/or protein expression levels of *aqp1*/Aqp1 and *aqp3*/Aqp3 in the gills of *P. annectens*, and that these changes could vary during the induction, maintenance, and arousal phases of aestivation.

## Materials and methods

### Animals

Specimens of *P. annectens* (80–150 g body mass) were imported from Central Africa through a local fish farm in Singapore. They were maintained in plastic aquaria filled with dechlorinated tap water at 25°C in the laboratory. Water was changed daily. No attempt was made to separate the sexes. Fish were acclimated to laboratory conditions for at least 2 weeks. During the acclimatization period, fish were fed with frozen fish meat. This study was performed in accordance with the approved protocol IACUC 035/09 granted by the Institutional Animal Care and Use Committee of the National University of Singapore.

### Experimental conditions and collection of samples

*P. annectens* kept in fresh water served as controls. After food was withheld for 96 h, control fish (*N* = 4) were killed with an overdose of neutralized 0.05% MS222 for tissue sampling. Lungfish were induced to aestivate at 27–29°C and 85–90% humidity individually in plastic tanks (L29 × W19 × H17.5 cm) containing 15 ml dechlorinated tap water (made Salinity = 0.3 with seawater), following the procedure of Chew et al. (2004). It took ~6 days for the lungfish to be encased in a brown dried mucus cocoon. In this study, these 6 days were counted as part of the aestivation period. The lungfish were allowed to aestivate for 6 months. In order to maintain a high humidity (>90%) within the tank, 1–2 ml of water was sprayed onto the side of the tank daily. After 6 months of aestivation, some lungfish were aroused by adding 200 ml of water into the tank and breaking up the cocoon manually. After a few minutes, the lungfish would swim sluggishly in the water; another 800 ml of water was added to cover the fish. Some lungfish were killed with a strong blow to the head for tissue sampling after 6 days (the induction phase), or after 6 months (the maintenance phase) of aestivation (*N* = 4 for each group). Others were killed with an overdose of neutralized 0.05% MS222 after 1 or 3 days of arousal from 6 months of aestivation (the arousal phase) without food (*N* = 4 for each group). The gills were quickly excised and freeze-clamped with aluminum tongs pre-cooled in liquid nitrogen.

### Total RNA extraction and cDNA synthesis

The total RNA was extracted from a gill sample using Tri Reagent™(Sigma-Aldrich Co., St. Louis, MO, USA), and purified using the Qiagen RNeasy Mini Kit (Qiagen GmbH, Hilden, Germany). RNA was quantified spectrophotometrically using a BioSpec-nano (Shimadzu, Tokyo, Japan) and RNA integrity assessed electrophoretically before storing at −80°C. A ratio 28S/18S rRNA was used to gauge the RNA integrity, while the absorbance ratio of A_260_/A_280_ was used to assess the purity of RNA and a ratio of ~2.0 was regarded as highly purified RNA. First strand cDNA was synthesized from 4 μg of total RNA using oligo(dT)_18_ primer and the RevertAid™ first strand cDNA synthesis kit (Fermentas International Inc., Burlington, ON, Canada).

### Polymerase chain reaction (PCR)

Partial sequences of *aqp1* and *aqp3* were obtained from the gills of *P. annectens* using gene-specific primers (Table 1). PCR was performed using Dreamtaq polymerase (Fermentas International Inc.), according to the manufacturer's instructions. The thermal cycling conditions were 95°C for 3 min, followed by 40 cycles of 95°C for 30 s, 60°C for 30 s, 72°C for 2 min, and a final extension of 72°C for 10 min. PCR products were separated by gel electrophoresis and the band of the correct molecular mass was excised and purified by FavorPrep™ Gel Purification Mini Kit (Favorgen Biotech Corp., Ping Tung, Taiwan) according to the manufacturer's instructions. Sequencing was performed using BigDye® Terminator v3.1 Cycle Sequencing Kit (Life Technologies Corporation, Carlsbad, California) and sequenced using the 3130XL Genetic Analyzer (Life Technologies Corporation).

**Table 1:** **Primers used for PCR, RACE, and qPCR of *aquaporin 1 (aqp1)* and *aquaporin 3 (aqp3)* from the gills of *Protopterus annectens***.

**Gene**	**Primer type**	**Primer sequence (5′to 3′)**
*aqp1*	PCR	Forward: ACCAAAGCAAGCTCTTCTG
		Reverse: GGATTCATTCCACAGCCA
	5′-RACE	GGACACCACAGAACCCAGCATTTGAG
	3′-RACE	GTTCTGTGGTGTCCGCTTCTATTCTG
	qPCR	Forward: CAGTATTAGTTCAGCAGTGGG
		Reverse: TCACTAACAGTCCAAGGGTC
*aqp3*	PCR	Forward: CTTGTGATGTTTGGCTGTG
		Reverse: AATCCAACAGTGAATGCCT
	5′-RACE	GTCCCATTGAAAGACCGATGACCAG
	3′-RACE	GGATTCTTTGATCAGCTCATTGGCACAG
	qPCR	Forward: TTTGCTTTATGCCTACTTGGTC
		Reverse: TGTTGAATTAGCTCCTGTTACTC

### Rapid amplification of cDNA ends (RACE)

Total RNA (1 μg) isolated from the gills of *P. annectens* was reverse transcribed into 5′-RACE-Ready cDNA and 3′-RACE-Ready cDNA using SMARTer™ RACE cDNA Amplification kit (Clontech Laboratories, Mountain View, CA, USA). RACE-PCR was performed using Advantage® 2 PCR kit (Clontech Laboratories), with gene-specific RACE primers (Table 1), designed based on partial cDNA sequences of *aqp1* and *aqp3* to generate the 5′ and 3′ cDNA fragments, respectively. The cycling conditions were 30 cycles of 94°C for 30 s, 65°C for 30 s, and 72°C for 4 min. RACE-PCR products were separated using gel electrophoresis, purified, and sequenced. Multiple sequencing was performed in both directions to obtain the full coding sequence. Sequence assembly and analysis were performed using Bioedit v7.1.3 (Hall, 1999). The complete coding cDNA sequences of *aqp1* and *aqp3* obtained from the gills of *P. annectens* have been deposited into Genbank with accession number KX494980 and KX494981, respectively.

### Deduced amino acid sequences and dendrogramic analyses

The Aqp1 and Aqp3 amino acid sequences were deduced from the corresponding nucleotide sequence using the ExPASy Proteomic server (http://web.expasy.org/translate/). The amino acid sequence was aligned and compared with selected Aqp1/AQP1 or Aqp3/AQP3 from various animal species using BioEdit. The transmembrane domains of Aqp1 or Aqp3 of *P. annectens* were identified using MEMSAT3 & MEMSAT-SVM provided by PSIPRED protein structure prediction server (http://bioinf.cs.ucl.ac.uk/psipred/; McGuffin et al., 2000). Potential phosphorylation sites were identified using NetPhos 2.0, and potential *N*-glycosylation sites were identified using NetNGlyc 1.0.

The sequences of Aqp1 and Aqp3 were aligned using ClustalX2, and the dendrograms of Aqp1/AQP1 and Aqp3/AQP3 were constructed through maximum likelihood analyses. Using ModelGenerator v0.85 (Keane et al., 2006), LG (Le and Gascuel, 2008) model was determined to be the best-fitting evolutionary model under the Akaike Information Criterion for the dendrograms of Aqp1/AQP1 and Aqp3/AQP3. The maximum likelihood analyses were run using RaxML v8.2.5 (Stamatakis, 2014) with 1000 bootstraps. Trees were determined to have converged after 500 and 650 replicates by the bootstrap convergence criterion for the dendrogram of Aqp1/AQP1 and Aqp3/AQP3, respectively. The accession numbers of selected amino acid sequences of Aqp1/AQP1 and Aqp3/AQP3 (from GenBank) used in the dendrogramic analysis are presented in Tables S1 and S2.

### Gene expression in various tissue/organs

PCR was performed to determine qualitatively the mRNA expression of *aqp1* or *aqp3* in the eye, brain, gill, heart, liver, spleen, pancreas, gut, kidney, lung, muscle, and skin of *P. annectens* using gene-specific qPCR primers (Table 1). Each PCR was carried out in a total volume of 10 μl using Dreamtaq polymerase (Fermentas International Inc.) with thermal cycling conditions: 95°C for 3 min, followed by 30 cycles of 95°C for 30 s, 55°C for 30 s, 72°C for 30 s, and a final extension of 72°C for 10 min. PCR products were then separated by electrophoresis in 2% agarose gel.

### qPCR

The method of absolute quantification with reference to a standard curve was adopted in this study, as it was essential to compare the mRNA expression levels of *aqp1* and *aqp3* in the gills of *P. annectens*. While relative quantitation methods produce fold-change data, they do not facilitate the comparison of gene expression levels. Moreover, relative quantification requires the incorporation of a reference gene, the expression of which is unaffected by the experimental conditions, but we had difficulties in identifying such a gene as substantial transcriptional changes occur in the gills of *P. annectens* during the three phases of aestivation.

RNA (4 μg) from the gills of *P. annectens* were extracted using the Qiagen RNeasy Plus Mini Kit (Qiagen GmbH). The sample was passed through gDNA Eliminator spin column provided by the Kit to remove genomic DNA. The purified RNA was subsequently reverse-transcribed using random hexamer primers with RevertAid™ first strand cDNA synthesis kit (Thermo Fisher Scientific, Waltham, MA, USA). To determine the absolute quantity of transcripts of *aqp1* and *aqp3* in a qPCR reaction, efforts were made to produce a pure amplicon (standard) of a defined region of the cDNA, as defined by the gene-specific set of qPCR primers (Table 1), from the gills of *P. annectens* following the method of Gerwick et al. (2007). PCR was performed with a specific set of qPCR primers and cDNA as a template in a final volume of 25 μl with the following cycling conditions: initial denaturation 95°C for 3 min, followed by 40 cycles of 95°C for 30 s, 60°C for 30 s, and 72°C for 30 s and 1 cycle of final extension of 72°C for 10 min. The PCR product was separated in a 2% agarose gel then excised and purified by FavorPrep™ Gel Purification Mini Kit (Favorgen Biotech Corp.) according to the manufacturer's instructions. The nucleotide fragments in the purified product were cloned using pGEM®-T Easy vector (Promega Corporation, Madison, WI, USA). The presence of the insert in the recombinant clones was confirmed by sequencing, and the cloned circular plasmid was quantified using a BioSpec-nano (Shimadzu).

The standard cDNA (template) was serially diluted (from 10^6^ to 10^2^ specific copies per 2 μl). qPCR was performed in triplicates using a StepOnePlus™ Real-Time PCR System (Life Technologies Corporation). The mRNA expression levels of *aqp1* and *aqp3* were determined using gene-specific qPCR primers (Table 1). The qPCR reactions contained 5 μl of KAPA SYBR® FAST Master Mix (2X) ABI Prism™(Kapa Biosystems, Woburn, MA, USA), 0.3 μmol l^−1^ of forward and reverse qPCR primers each and 1 ng of sample cDNA or various quantities of standard in a total volume of 10 μl. Cycling conditions were 95°C for 20 s (1 cycle), followed by 40 cycles of 95°C for 3 s and 62°C for 30 s. Data (C_*t*_values) were collected at each elongation step. A melt curve analysis was performed after each run by increasing the temperature from 60 to 95°C in 0.3°C increments to confirm the presence of a single product only. The PCR products obtained were also separated in a 2% agarose gel to verify the presence of a single band. A standard curve was obtained from plotting threshold cycle (C_*t*_) on the Y-axis and the natural log of concentration (copies μl^−1^) on the X-axis. The C_*t*_ slope, PCR efficiency, Y-intercept, and correlation coefficient (*r*^2^) were calculated using the default setting of StepOne Software v2.1 (Life Technologies Corporation). Diluted standards were stored at −20°C. The PCR efficiencies for *aqp1* and *aqp3* were 96.5 and 93.2%, respectively. The quantity of transcript in an unknown sample was determined from the linear regression line derived from the standard curve and expressed as copies of transcripts per ng total RNA.

### SDS-PAGE electrophoresis and western blotting

A commercial firm (GenScript, Piscataway, NJ, USA) was engaged to raise a rabbit polyclonal antibody against aa 240–253 (AFTGGNVEEYDLDG) of Aqp1 and a rabbit polyclonal antibody against aa 211–224 (GYAVNPARDLGPRV) of Aqp3 from the gills of *P. annectens*. Immunoreactive bands of Aqp1 and Aqp3 were visualized at the expected molecular mass of 28.6 and 31.7 kDa, respectively.

Western blotting was performed on the gills obtained from the control fish and fish that had undergone 6 days, or 6 months of aestivation, or 1 or 3 days of arousal from 6 months of aestivation. Individual samples were homogenized twice in five volumes (w/v) of ice cold buffer containing 50 mmol l^−1^ Tris HCl, (pH 7.4), 1 mmol l^−1^ EDTA, 150 mmol l^−1^ NaCl, 1 mmol l^−1^ NaF, 1 mmol l^−1^ Na_3_VO_4_, 1% NP-40, 1% sodium deoxycholate, 1 mmol l^−1^ phenylmethylsulfonyl fluoride, and 1 × HALT protease inhibitor cocktail (Thermo Fisher Scientific) using pre-cooled TissueLyser LT (Qiagen GmbH) for 2.5 min at 50 Hz. The homogenate was centrifuged at 10,000 × *g* for 20 min at 4°C. The protein concentration in the supernatant obtained was determined according to the method of Bradford (1976) and adjusted to 10 μg μl^−1^ with Laemmli buffer (Laemmli, 1970). Samples were heated at 70°C for 15 min, and then kept at −80°C until analysis.

Proteins were separated by SDS-PAGE (12% acrylamide for resolving gel, 4% acrylamide for stacking gel) according to the method of Laemmli (1970) using a vertical mini-slab apparatus (Bio-Rad Laboratories, Hercules, CA, USA). The amount of protein loaded for gel separation was 40 μg for Aqp1 and 100 μg for Aqp3. Proteins were then electrophoretically transferred onto PVDF membranes using a transfer apparatus (Bio-Rad Laboratories). The concentrations of the anti-Aqp1 and anti-Aqp3 antibodies were 0.8 and 1.7 μg ml^−1^, respectively. Peptide competition assay was performed to validate the specificity of the anti-Aqp1 and anti-Aqp3 antibodies. The anti-Aqp1 (8 μg) and anti-Aqp3 (16.7 μg) antibodies were pre-incubated with the immunizing peptide of Aqp1 (40 μg) and Aqp3 (83.5 μg) provided by GenScript (Piscataway, NJ, USA), respectively, in a total volume of 200 μl for 1 h at 25°C and subsequently used for the peptide competition assay. Detection was performed using Pierce SuperSignal West Pico Rabbit Fast Western Kit (Thermo Fisher Scientific), according to manufacturer's instruction. Bands were visualized by chemiluminescence (Western Lightning, PerkinElmer Life Sciences, Boston, MA, USA) using X-ray film (Thermo Fisher Scientific) which were processed by a Kodak X-Omat 3000 RA processor (Kodak, Rochester, NY, USA). The blots were scanned using a CanonScan 4400F flatbed scanner in TIFF format at 300 dpi resolution. Densitometric quantification of the band intensities was performed using ImageJ (version 1.40, NIH), calibrated with a calibrated 37 step reflection scanner scale (1″ × 8″; Stouffer #R3705-1C). Difficulties were encountered in finding a reference protein, the expression of which would be unaffected throughout the three phases of aestivation. Hence, results were expressed as arbitrary densitometric unit per μg protein, i.e., with reference to the total protein abundance, as reported elsewhere for several other proteins from *P. annectens* during aestivation (Ching et al., 2014; Hiong et al., 2014, 2015; Ong et al., 2015).

### Statistical analyses

Results were presented as means ± standard errors of the mean (S.E.M.). Statistical analyses were performed using SPSS version 18 (SPSS Inc, Chicago, USA). Homogeneity of variance was checked using Levene's Test. Differences between means were tested using one-way analysis of variance followed by multiple comparisons of means by Tukey *post-hoc* test, depending on the homogeneity of variance of the data set. Differences with *P* < 0.05 were reported as statistically significant.

## Results

### The nucleotide and the deduced amino acid sequences of *aqp1*/Aqp1 and *aqp3*/Aqp3

**Figure 1:**
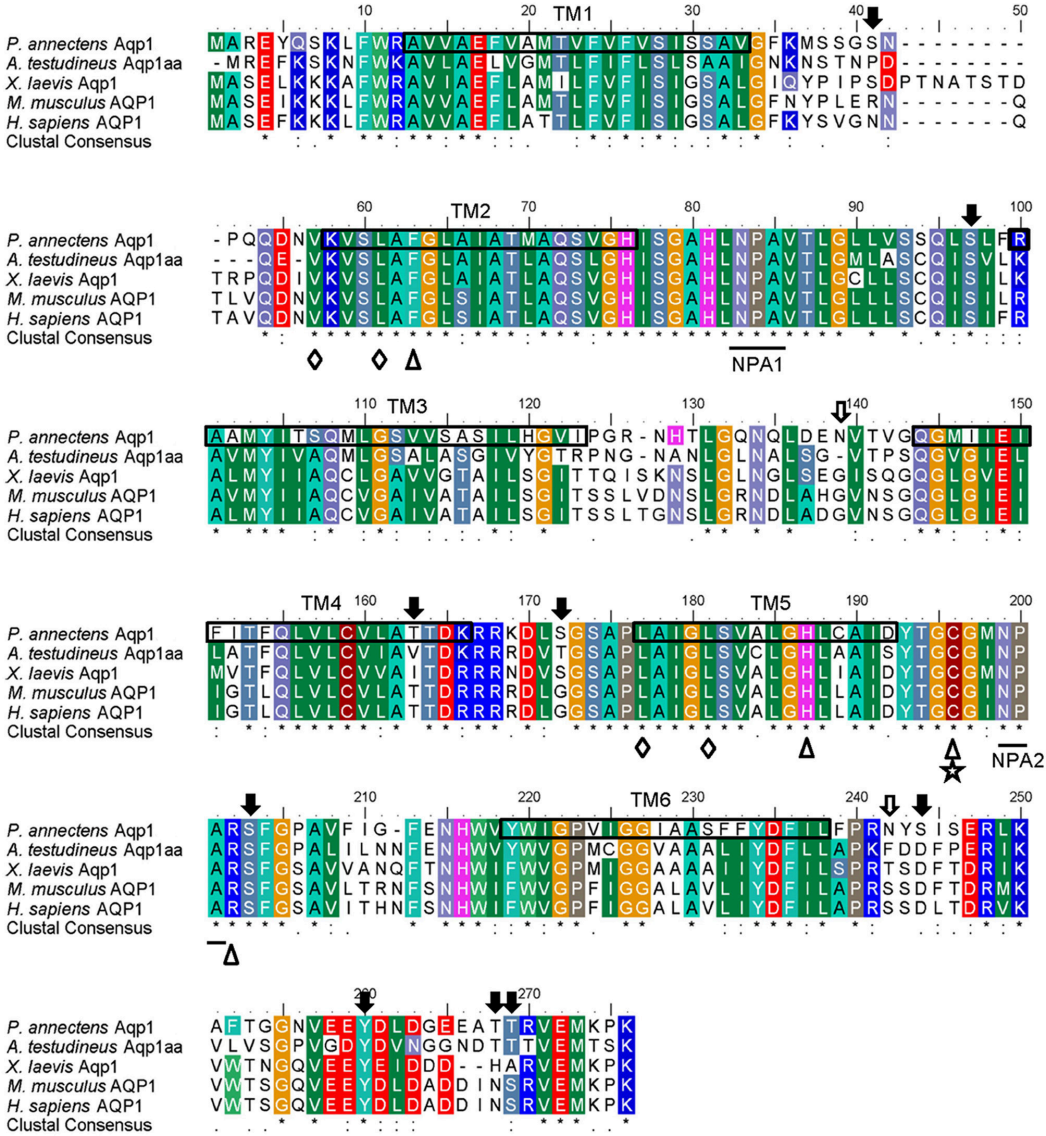
**A multiple amino acid alignment of aquaporin 1 (Aqp1) from *Protopterus annectens* with *Anabas testudineus* Aqp1aa (AGF30363.1), *Xenopus laevis* Aqp1 (NP_001085391.1), *Mus musculus* AQP1 (EDK98728.1), and *Homo sapiens* AQP1 (CAQ51480.2)**. Identical amino acid residues are indicated by asterisks, strongly similar amino acids are indicated by colons and weakly similar amino acids are indicated by periods. Substrate discrimination residues at the aromatic/arginine (ar/R) constriction site are indicated with open triangles. Central pore-lining residues are indicated with open diamonds. The binding site for AQP1-inhibitor HgCl_2_ is indicated by a five-point star. The asparagine–proline–alanine (NPA) motifs are underlined. Potential *N*-glycosylation and phosphorylation sites are indicated by open and shaded arrows, respectively. The predicted transmembrane domains (TM1–6) of Aqp1 of *P. annectens* are indicated by open boxes and were predicted using MEMSATS & MEMSAT-SVA provided by PSIPRED protein structure prediction server.

The complete coding cDNA sequence of *aqp1* obtained from the gills of *P*. *annectens* consisted of 798 bp, coding for 265 amino acids with an estimated molecular mass of 28.6 kDa. The nucleotide sequence of *aqp1* obtained from the gills (KX494980) is identical to *aqp1* obtained by Konno et al. (2010) presumably from the kidney, lung and eye (Accession: AB499798.1) of *P. annectens*. The deduced amino acid sequence of Aqp1 of *P. annectens* had six transmembrane domains (Figure 1). An alignment of Aqp1 of *P. annectens* with Aqp1/AQP1 from human, mouse, *Xenopus laevis* and *Anabas testudineus* indicated that the asparagine–proline–alanine (NPA) motifs, the substrate discriminating residues at the aromatic/arginine (ar/R) constriction site (F63. H187, C196, and R202) and the central pore-lining residues (V57, L61, L177, and L181) were highly conserved (Figure 1). Nine potential phosphorylation sites and two *N*-glycosylation sites were identified in Aqp1 in *P. annectens* (Figure 1). Aqp1 of *P. annectens* shared the highest sequence similarity with mammalian AQP1 (60.6–64.8%), followed by amphibian Aqp1 (53.4–60.5%), actinopterygian Aqp1/Aqp1a (51.6–58.3%), *L. chalumnae* Aqp1 (57.6%), and actinopterygian Aqp1b (49.0–53.3%, Table 2).

**Table 2:** **The percentage similarity, arranged in a descending order, between the deduced amino acid sequence of aquaporin 1 (Aqp1) from *Protopterus annectens* and Aqp1/AQP1 sequences from other animal species obtained from GenBank**.

**Classification**	**Species (accession number)**	**Similarity (%)**
Mammals	*Homo sapiens* AQP1 (CAQ51480.2)	64.8
	*Pongo abelii* AQP1 (NP_001126220.1)	64.8
	*Mus musculus* AQP1 (EDK98728.1)	63.7
	*Rattus norvegicus* AQP1 (EDL88090.1)	63.3
	*Sus scrofa* AQP1 (NP_999619.1)	62.7
	*Canis lupus familiaris* AQP1 (NP_001003130.1)	61.9
	*Bos taurus* AQP1 (ABF57368.1)	61.2
	*Ovis aries* AQP1 (AAB63463.1)	60.6
Amphibians	*Xenopus laevis* Aqp1 (NP_001085391.1)	60.5
	*Xenopus (Silurana) tropicalis* Aqp1 (NP_001005829.1)	60.5
	*Rhinella marina* Aqp1 (AAA67782.1)	53.4
Actinopterygians	*Takifugu obscurus* Aqp1 (ADG86337.1)	58.3
	*Osmerus mordax* Aqp1 (ACO09149.1)	58.1
	*Poecilia formosa* Aqp1 (XP_007548683.1)	57.9
	*Anabas testudineus* Aqp1aa (AGF30363.1)	57.9
	*Acanthopagrus schlegelii* Aqp1 (ABO38816.1)	57.9
	*Rhabdosargus sarba* Aqp1 (AEG78286.1)	57.9
	*Diplodus sargus* Aqp1 (AEU08496.1)	57.9
	*Sparus aurata* Aqp1a (ABM26907.1)	57.9
	*Dicentrarchus labrax* Aqp1 (ABI95464.2)	57.6
	*Fundulus heteroclitus* Aqp1 (ACI49538.1)	57.6
	*Danio rerio* Aqp1 (ACA29537.1)	56.7
	*Anguilla japonica* Aqp1a (BAC82109.1)	56.5
	*Oryzias dancena* Aqp1 (BAN17349.1)	56.1
	*Anguilla anguilla* Aqp1a (CAD92027.1)	56.1
	*Cynoglossus semilaevis* Aqp1 (ADG21868.1)	55.7
	*Danio rerio* Aqp1a (NP_996942.1)	55.5
	*Cyprinus carpio* Aqp1a1-1 (BAS18938.1)	55.2
	*Anguilla anguilla* Aqp1b (ABM26906.1)	53.3
	*Salmo salar* Aqp1b (NP_001133472.1)	53.1
	*Anguilla japonica* Aqp1b (BAK53383.1)	52.9
	*Oryzias latipes* Aqp1 (XP_011485314.1)	51.6
	*Sparus aurata* Aqp1b (ABM26908.1)	49.0
Sarcopterygian	*Latimeria chalumnae* Aqp1 (XP_006005961.1)	57.6

**Figure 2:**
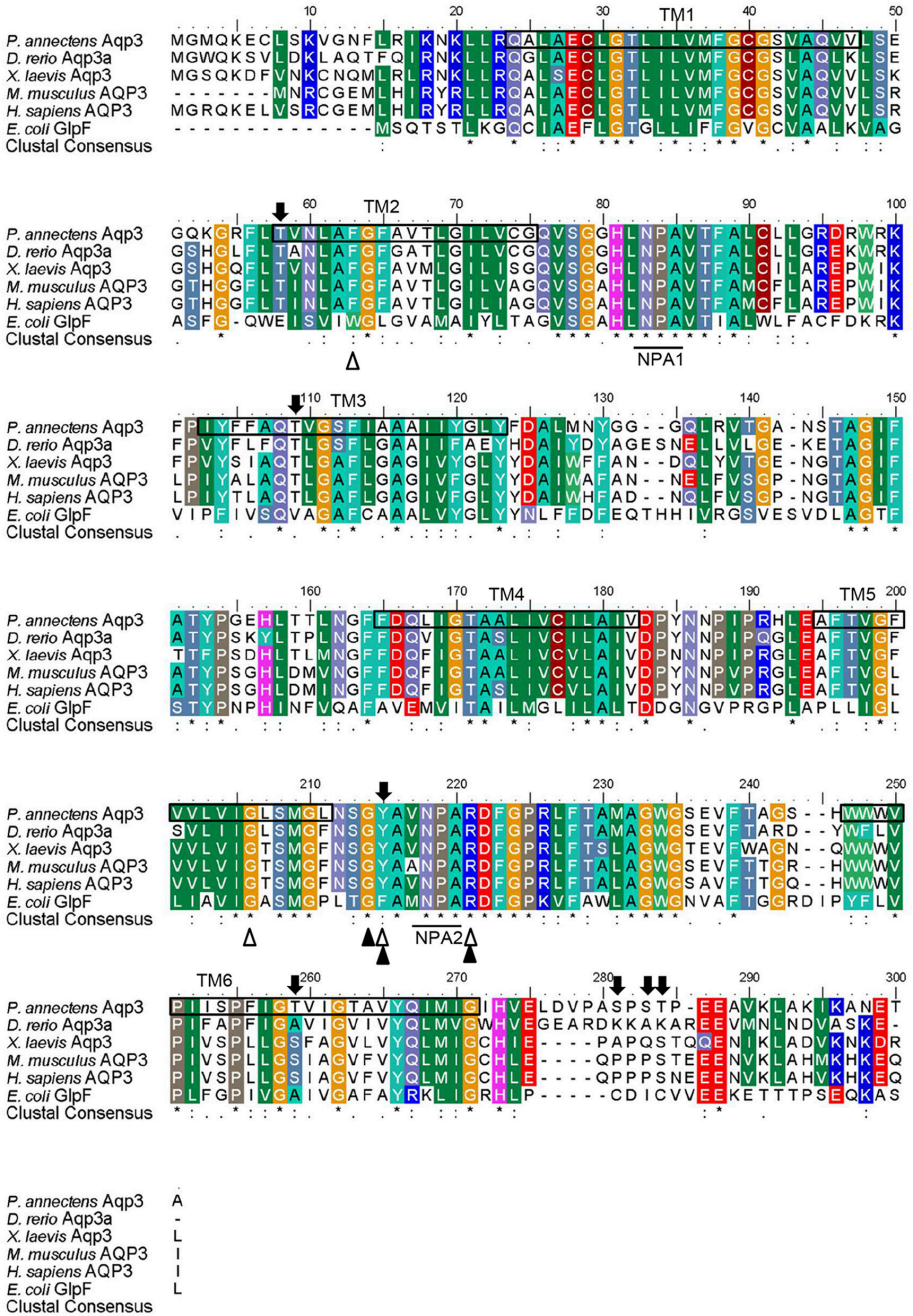
**A multiple amino acid alignment of aquaporin 3 (Aqp3) from *Protopterus annectens* with *Danio rerio* Aqp3a *(*AAH44188.1), *Xenopus laevis* Aqp3 (NP_001081876.1), *Mus musculus* AQP3 (BAB03270.1), *Homo sapiens* AQP3 (AAY68214.1), and *Escherichia coli* glycerol facilitator (GlpF; BAB03270.1)**. Identical amino acid residues are indicated by asterisks, strongly similar amino acids are indicated by colons and weakly similar amino acids are indicated by periods. Substrate discrimination residues at the aromatic/arginine (ar/R) constriction site are indicated with open triangles. Glycerol binding sites are indicated with shaded triangles. The asparagine–proline–alanine (NPA) motifs are underlined. Potential phosphorylation sites are indicated by shaded arrows. The predicted transmembrane domains (TM1–6) of Aqp3 of *P. annectens* are indicated by open boxes and were predicted using MEMSATS & MEMSAT-SVA provided by PSIPRED protein structure prediction server.

The complete coding cDNA sequence of *aqp3* obtained from the gills of *P*. *annectens* consisted of 888 bp, coding for 295 amino acids with an estimated molecular mass of 31.7 kDa. The similarity between this *aqp3* nucleotide sequence from the gills (KX494981) and that reported by Konno et al. (2010) for other organs (Accession: AB499799.1) is 99.8%, with guanine being replaced by adenine at position 594 in the *aqp3* obtained in this study. A hydropathy analysis revealed that the deduced amino acid sequence of Aqp3 of *P. annectens* had six transmembrane domains (Figure 2). Efforts were made to compare Aqp3 of *P. annectens* with *Escherichia coli* glycerol facilitator (GlpF), as its high resolution crystal structure had been resolved and based on which theories of water and glycerol conduction by aquaglyceroporins had been formulated (Fu et al., 2000; Jensen et al., 2001). An alignment of Aqp3 of *P. annectens* with *E. coli* GlpF and Aqp3/AQP3 from human, mouse, *X. laevis* and *Danio rerio* revealed highly conserved NPA motifs (Figure 2). The residues at the ar/R constriction site (F63. G214, Y215, and R221) and glycerol binding sites (G214, Y215, and R221) were conserved in Aqp3 of *P. annectens* as compared to Aqp3/AQP3 from human, mouse, *X. laevis*, and *D. rerio* (Figure 2). There were seven potential phosphorylation sites in Aqp3 in *P. annectens* (Figure 2). Aqp3 of *P. annectens* shared the highest sequence similarity with amphibian Aqp3 (69.1–70.1%), followed by mammalian AQP3 (67.7–69.8%), actinopterygian Aqp3 (62.0–68.8%), and *L. chalumnae* Aqp3 (68.4%; Table 3).

**Table 3:** **The percentage similarity, arranged in a descending order, between the deduced amino acid sequence of aquaporin 3 (Aqp3) from *Protopterus annectens* and Aqp3/AQP3 sequences from other animal species obtained from GenBank**.

**Classification**	**Species (accession number)**	**Similarity (%)**
Amphibians	*Xenopus (Silurana) tropicalis* Aqp3 (CAJ82459.1)	70.1
	*Xenopus laevis* Aqp3 (NP_001081876.1)	69.1
Mammals	*Pan troglodytes* AQP3 (JAA01153.1)	69.8
	*Homo sapiens* AQP3 (AAY68214.1)	69.4
	*Bos taurus* AQP3 (NP_001073262.1)	69.4
	*Sus scrofa* AQP3 (ABW06862.1)	69.4
	*Rattus norvegicus* AQP3 (EDL98656.1)	69.4
	*Macaca mulatta* AQP3 (NP_001244972.1)	69.1
	*Mus musculus* AQP3 (BAB03270.1)	68.1
	*Rattus rattus* AQP3 (BAA04559.1)	67.7
Actinopterygians	*Anguilla japonica* Aqp3 (BAH89253.1)	68.8
	*Danio rerio* Aqp3 (AAH44188.1)	68.5
	*Takifugu rubripes* Aqp3 (XP_003975282.1)	68.3
	*Larimichthys crocea* Aqp3 (KKF15034.1)	68.1
	*Anguilla anguilla* Aqp3 (CAC85286.1)	67.5
	*Ictalurus punctatus* Aqp3 (AHH37605.1)	67.5
	*Oryzias dancena* Aqp3 (BAP11294.1)	67.3
	*Dicentrarchus labrax* Aqp3 (ABG36519.1)	67.1
	*Takifugu obscurus* Aqp3 (ADG86338.1)	66.9
	*Poecilia reticulata* Aqp3 (XP_008417140.1)	66.8
	*Poecilia formosa* Aqp3 (XP_007562721.1)	66.8
	*Danio rerio* Aqp3b (NP_001159593.1)	66.8
	*Tribolodon hakonensis* Aqp3 (BAB83082.1)	66.5
	*Notothenia coriiceps* Aqp3 (XP_010776959.1)	66.4
	*Oryzias latipes* Aqp3 (XP_004072505.1)	66.3
	*Sarotherodon melanotheron* Aqp3 (AHA92968.1)	65.7
	*Fundulus heteroclitus* Aqp3 (NP_001296892.1)	65.4
	*Oreochromis niloticus* Aqp3 (AHY84681.1)	64.8
	*Oreochromis mossambicus* Aqp3 (BAD20708.1)	64.8
	*Maylandia zebra* Aqp3 (XP_004544455.1)	64.8
	*Cynoglossus semilaevis* Aqp3 (XP_008323950.1)	62.0
Sarcopterygian	*Latimeria chalumnae* Aqp3 (XP_006004793.1)	68.4

### Dendrogramic analyses of Aqp1 and Aqp3

**Figure 3:**
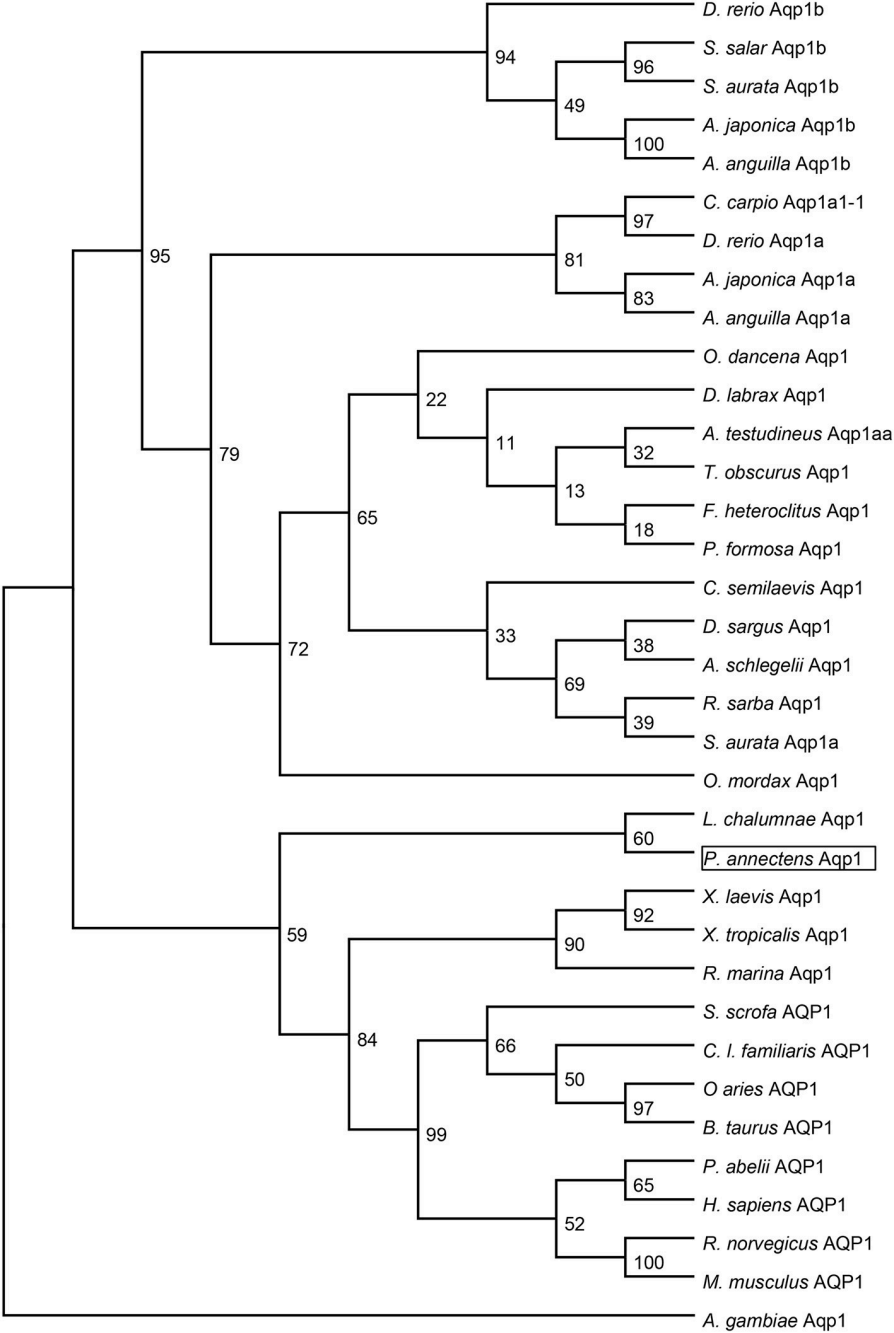
**A dendrogram of aquaporin 1 (Aqp1/AQP1) including Aqp1 of *Protopterus annectens***. The support for nodes is indicated by % bootstrap support (out of 1000) in the maximum likelihood analysis, employing the LG (Le and Gascuel, 2008) model of amino acid substitution. *Anopheles gambiae* Aqp1 is used as outgroup for the dendrogram.

**Figure 4:**
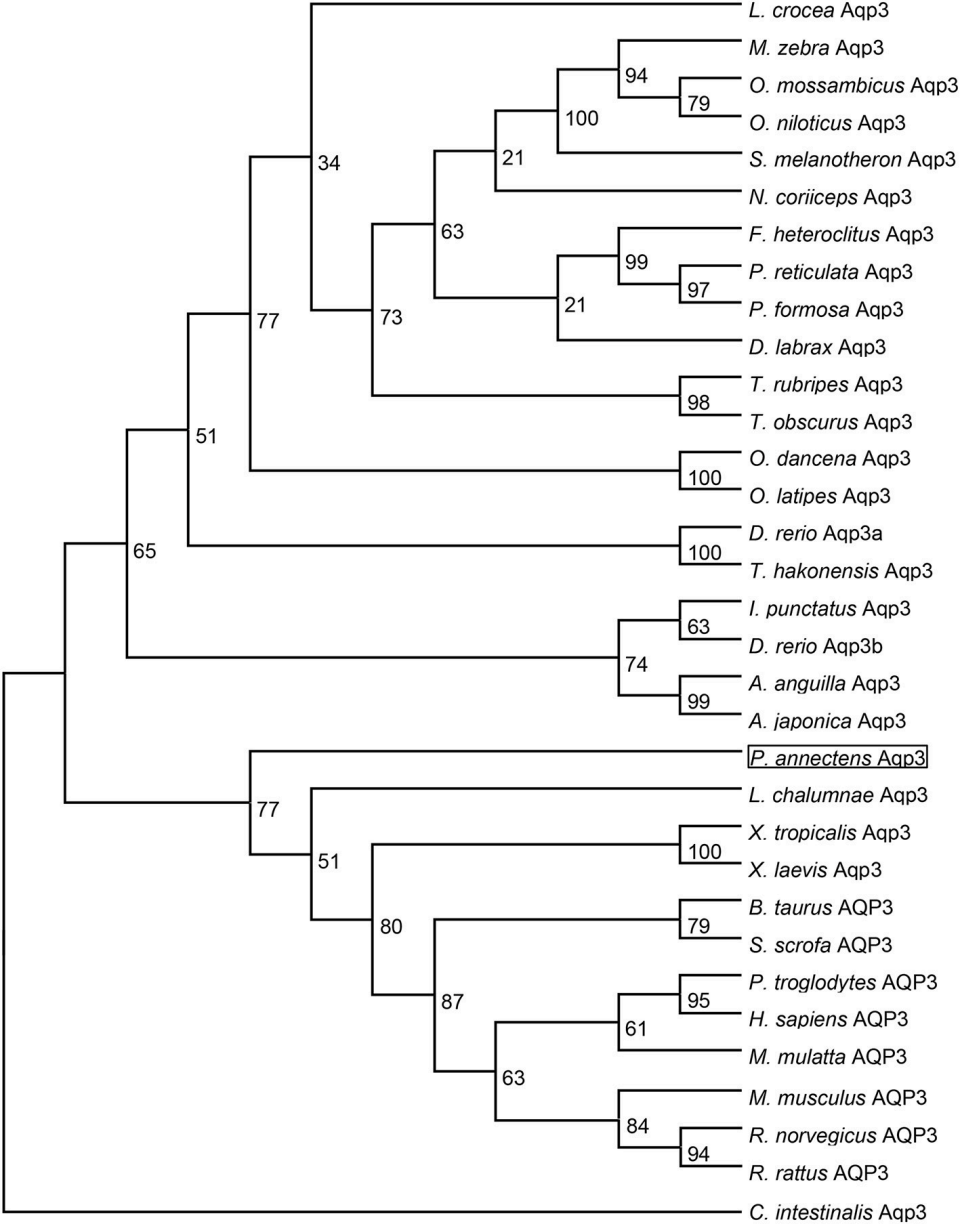
**A dendrogram of aquaporin 3 (Aqp3/AQP3) including Aqp3 of *Protopterus annectens***. The support for nodes is indicated by % bootstrap support (out of 1000) in the maximum likelihood analysis, employing the LG (Le and Gascuel, 2008) model of amino acid substitution. *Ciona intestinalis* Aqp3 is used as outgroup for the dendrogram.

Dendrogramic analyses of Aqp1 (Figure 3) and Aqp3 (Figure 4) of *P. annectens* indicated their close relationships with Aqp1 and Aqp3 of *Latimeria chalumnae*, respectively, and indicated that *P. annectens* was phylogenetically closer to tetrapods than to actinopterygians.

### The mRNA expression levels of *aqp1* and *aqp3* in various tissues/organs of *P. annectens*

**Figure 5:**
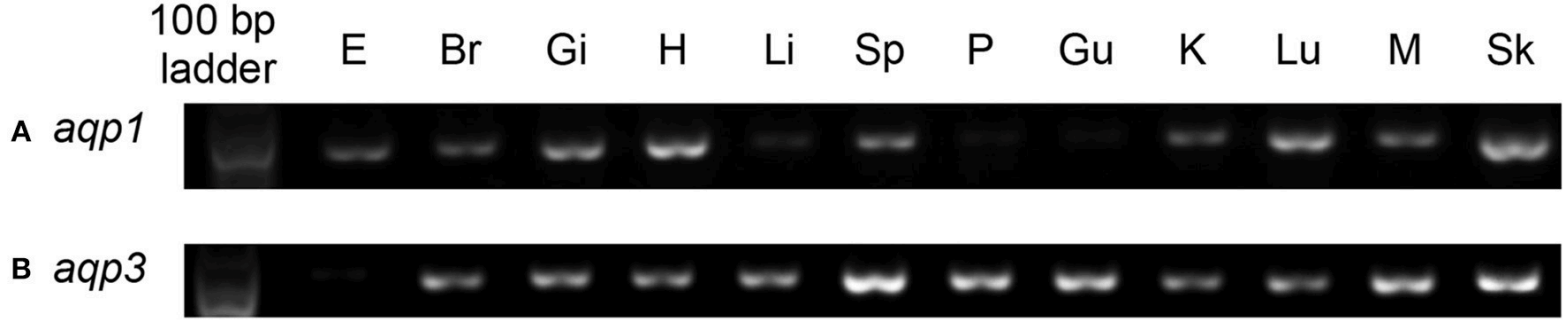
**The gene expression of (A)** *aquaporin 1 (aqp1)* and **(B)** *aquaporin 3 (aqp3)* in various tissues/organs of *Protopterus annectens*. The mRNA expression of *aqp1* and *aqp3* were examined in the eyes (E), brain (Br), gills (Gi), heart (H), liver (Li), spleen (Sp), pancreas (P), gut (Gu), kidney (K), Lung (Lu), muscle (M), and skin (Sk), of *Protopterus annectens* kept in fresh water.

The expression of *aqp1* was detected strongly in the gills, heart, lung and skin, but weakly in the eyes, brain, spleen, kidney, and muscle of *P. annectens* kept in fresh water (Figure 5A). The expression of *aqp3* was detected in all the organs/tissues examined, except the eye (Figure 5B).

### Effects of aestivation on the mRNA expression levels of *aqp1* and *aqp3* in the gills

**Figure 6:**
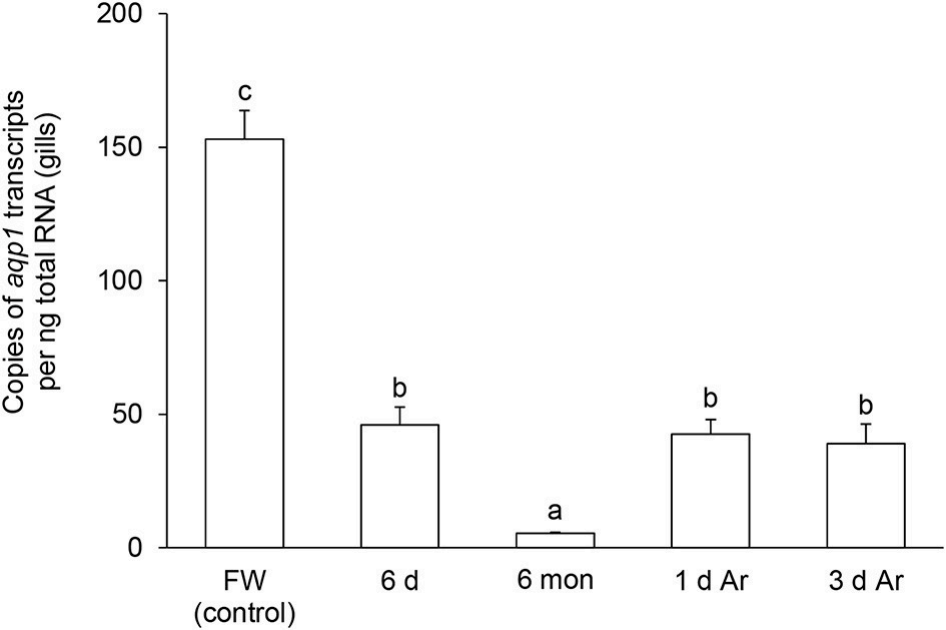
**The absolute quantification of mRNA expression level (copies of transcript per ng total RNA) of *aquaporin 1* (*aqp1*) in the gills of *Protopterus annectens* kept in fresh water on day 0 (FW; control), after 6 days (d; induction phase) or 6 months (mon; maintenance phase) of aestivation, or after 1 or 3 d of arousal (Ar) from 6 mon of aestivation**. Results represent means ± S.E.M (*N* = 4). Means not sharing the same letter are significantly different (*P* < 0.05).

**Figure 7:**
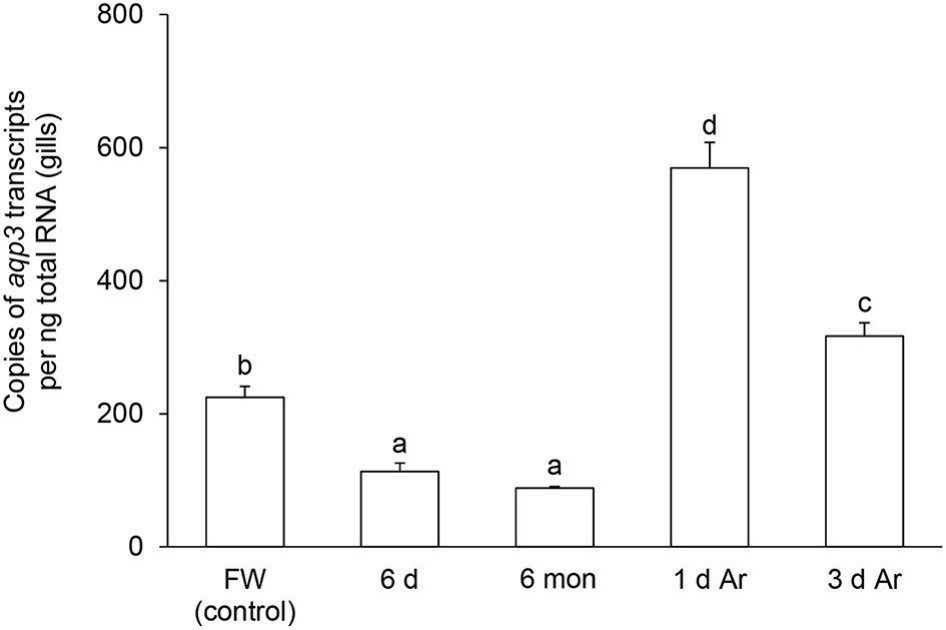
**The absolute quantification of mRNA expression level (copies of transcript per ng total RNA) of *aquaporin 3* (*aqp3*) in the gills of *Protopterus annectens* kept in fresh water on day 0 (FW; control), after 6 days (d; induction phase) or 6 months (mon; maintenance phase) of aestivation, or after 1 or 3 d of arousal (Ar) from 6 mon of aestivation**. Results represent means ± S.E.M (*N* = 4). Means not sharing the same letter are significantly different (*P* < 0.05).

The transcript level of *aqp1* in the gills of *P. annectens* decreased significantly after 6 days (by 70%; *P* < 0.05), or 6 months (by 96%; *P* < 0.05) of aestivation, or after 1 day (by 72%; *P* < 0.05), or 3 days (by 75%; *P* < 0.05) of arousal from 6 months of aestivation (Figure 6). As for *aqp3*, its transcript level decreased significantly in the gills of *P. annectens* after 6 days (by 50%; *P* < 0.05) or 6 months (by 61%; *P* < 0.05) of aestivation (Figure 7). However, there were significant increases in the mRNA expression levels of *aqp3* in the gills of *P. annectens* after 1 day (2.5-fold; *P* < 0.05) or 3 days (1.4-fold; *P* < 0.05) of arousal from 6 months of aestivation as compared with the control (Figure 7).

### Effects of aestivation on the protein abundance of Aqp1 and Aqp3 in the gills

**Figure 8:**
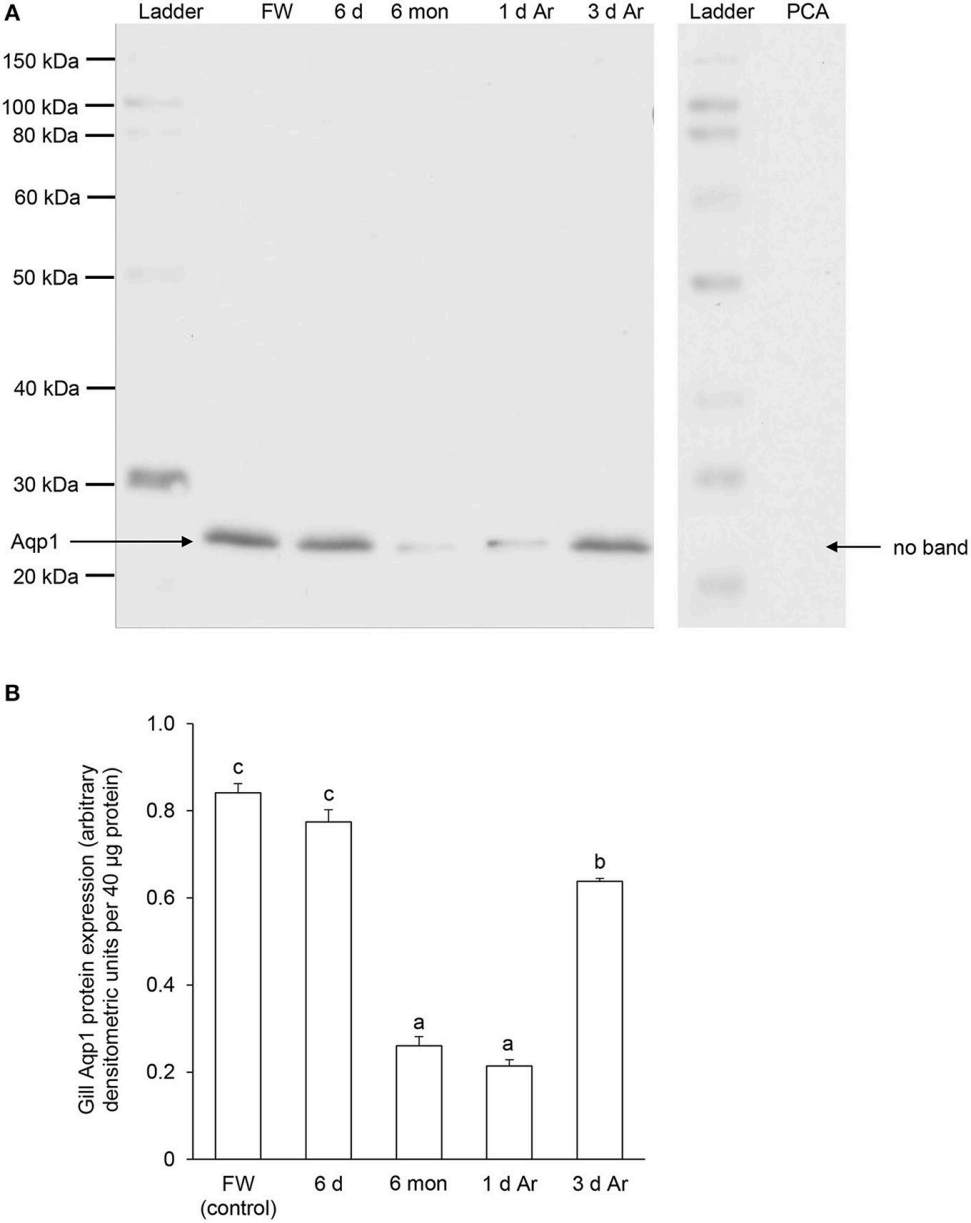
**The protein abundance of aquaporin 1 (Aqp1) in the gills of *Protopterus annectens* kept in fresh water on day 0 (FW; control), after 6 days (d; induction phase) or 6 months (mon; maintenance phase) of aestivation, or after 1 or 3 d of arousal (Ar) from 6 mon of aestivation. (A)** An example of immunoblot of Aqp1 (left) and Aqp1 preincubated with immunising peptide for the peptide competition assay (PCA; right). **(B)** The protein abundance of Aqp1 expressed as arbitrary densitometric units per 40 μg protein. Results represent mean ± S.E.M. (*N* = 3). Means not sharing the same letter are significantly different (*P* < 0.05).

Western blotting using the custom-made anti-Aqp1 antibody revealed a band at ~26 kDa which is close to the estimated molecular mass of the deduced Aqp1 sequence of *P. annectens*, and the validity of antibody binding was verified through a peptide competition test (Figure 8A). While the protein abundance of Aqp1 remained unchanged in the gills of *P. annectens* after 6 days of aestivation, it decreased significantly after 6 months (by 69%; *P* < 0.05) of aestivation (Figure 8B). The branchial protein abundance of Aqp1 of fish which had been aroused from aestivation for 1 day remained comparable to that of fish which had undergone 6 months of aestivation, but it partially recovered to ~75% of the control value by day 3 of arousal despite being significantly lower than that of the control (Figure 8B).

**Figure 9:**
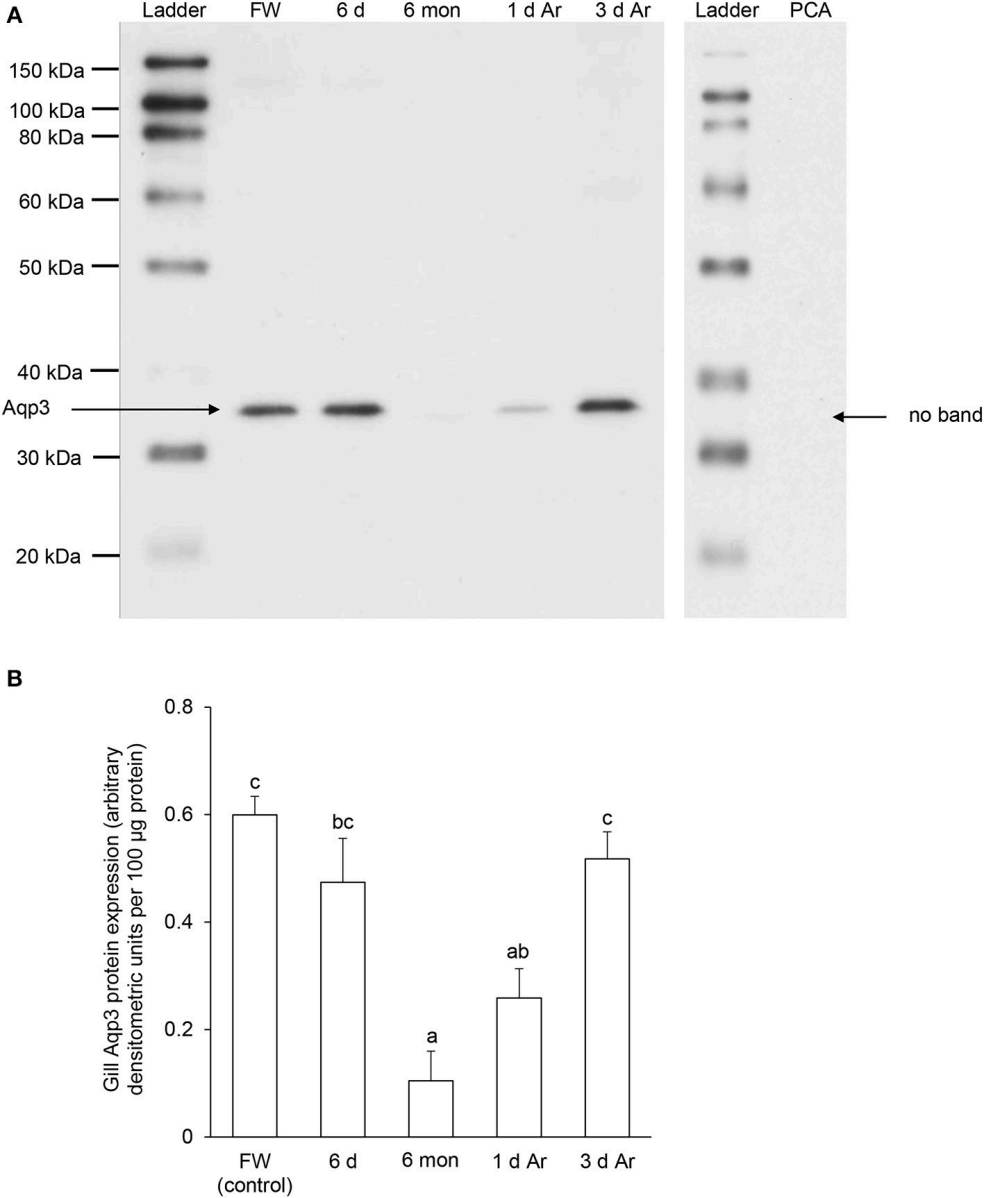
**The protein abundance of aquaporin 3 (Aqp3) in the gills of *Protopterus annectens* kept in fresh water on day 0 (FW; control), after 6 days (d; induction phase) or 6 months (mon; maintenance phase) of aestivation, or after 1 or 3 d of arousal (Ar) from 6 mon of aestivation. (A)** An example of immunoblot of Aqp3 (left) and Aqp3 preincubated with immunising peptide for the peptide competition assay (PCA; right). **(B)** The protein abundance of Aqp3 expressed as arbitrary densitometric units per 100 μg protein. Results represent mean ± S.E.M. (*N* = 3). Means not sharing the same letter are significantly different (*P* < 0.05).

For Aqp3, Western blotting revealed a band at ~36 kDa which is close to the estimated molecular mass of the deduced Aqp3 sequence of *P. annectens*, and the validity of antibody binding was verified through a peptide competition test (Figure 9A). The protein abundance of Aqp3 remained unchanged in the gills of *P. annectens* after 6 days of aestivation, but it decreased significantly after 6 months (by 83%; *P* < 0.05) of aestivation, or after 1 day (by 57%; *P* < 0.05) of arousal from 6 months of aestivation (Figure 9B). On day 3 of arousal, the protein abundance of branchial Aqp3 returned to the control level (Figure 9B).

## Discussion

### Molecular characterization of Aqp1 and Aqp3

Both Aqp1 and Aqp3 comprise two highly conserved NPA motifs which play a crucial role in water-selective permeation and cation/proton exclusion (Murata et al., 2000; Fu and Lu, 2007; Wree et al., 2011). An alignment of the Aqp1 sequence of *P. annectens* with those from other animal species also reveals highly conserved substrate discriminating residues at the ar/R constriction site and the central pore-lining residues. The substrate discriminating residues at the ar/R constriction site for Aqp1 of *P. annectens* consist of F63, H187, C196, and R202, which correspond to F56, H180, C189, and R195 of human AQP1. In human AQP1, C189 is the binding site for HgCl_2_, which is an AQP-inhibitor (Preston et al., 1993; Zhang et al., 1993), while H180 and R195 provide a hydrophilic edge with F56 (Sui et al., 2001). Together with the NPA motifs, they act as major sites for channel selectivity and proton exclusion (de Groot and Grubmüller, 2005; Wu and Beitz, 2007). In contrast to Aqp1, the substrate discriminating residues at the ar/R constriction site of Aqp3 comprise F63, G206, Y215, and R221, of which G206 and Y215 are conserved in Aqp3/AQP3 among human, mouse, frog, lungfish, and fish. In *E. coli* GlpF, the substrate discriminating residues at the ar/R constriction site consist of W48, G199, F200, and R206 (Klein et al., 2015), with G199, F200, and R206 constituting the glycerol-binding site (Stroud et al., 2003). The aromatic rings of W48 and F200 provide a hydrophobic corner to interact with the alkyl backbone of glycerol (Fu and Lu, 2007). The guanidinium group of R206 and the carbonyl oxygens of G199 and F200 on the opposing hydrophilic face provide hydrogen bonds for interactions with a pair of glycerol hydroxyls (Fu and Lu, 2007). It has been demonstrated that GlpF-W48F/F200T mutant renders the aquapore wider and more hydrophilic, thus increasing the occupancy and permeability of water (Tajkhorshid et al., 2002). Since W48 and F200 in *E. coli* GlpF are replaced with F63 and Y215, respectively, in Aqp3 of *P. annectens*, these replacements could potentially increase the pore size and hydrophilicity of the aquapore to enhance water permeation.

### Dendrogramic analyses of Aqp1 and Aqp3

Several molecular phylogenetic studies have denoted lungfishes as the closest living relatives of tetrapods (Takezaki et al., 2004; Hallström and Janke, 2009; Amemiya et al., 2013). Indeed, our dendrogramic analyses indicated that Aqp1 and Aqp3 of *P. annectens* were grouped closer to tetrapods than to teleost fishes, which corroborate previous reports on the evolution of Aqp/AQP (Finn et al., 2014; Finn and Cerdà, 2015). Furthermore, our results are in agreement with previous reports on genes/proteins of *P. annectens* that bear close relationship with those of tetrapods (argininosuccinate lyase, Chng et al., 2014; gulonolactone oxidase, Ching et al., 2014; Na^+^/K^+^-ATPase α-subunit isoforms, Hiong et al., 2014; betaine-homocysteine *S*-methyltransferase 1, Ong et al., 2015; coagulation factor II and fibrinogen gamma chain, Hiong et al., 2015).

### Decreases in *aqp1* and *aqp3* transcript levels, but not Aqp1 and Aqp3 protein abundance, in the gills during the induction phase of aestivation

During the induction phase of aestivation, the lungfish is on land and the gills are exposed to air. Due to the lack of water, the gills can no longer function effectively as a respiratory, excretory, and osmoregulatory organ. While hyperventilation occurs, the lungfish depends mainly on pulmonary rather than branchial respiration (DeLaney and Fishman, 1977; Lomholt, 1993; Laurent, 1996). As for nitrogenous waste excretion, the rates of ammonia and urea excretion decrease (Chew et al., 2004), presumably due to a lack of water to flush the branchial epithelium. Concerning osmo- and ion-regulation, the aestivating lungfish is not confronted with a constant gain of water and loss of ions in a hypoosmotic environment. However, it would lose water from the gill surface through evaporation leading to dehydration of branchial epithelial cells. Hence, it would be important for the lungfish to make the necessary preparation to shut down gill functions and to prevent cells from dehydration during the induction phase of aestivation. Indeed, there were significant decreases in the transcript levels of *aqp1* and *aqp3*, while the protein abundance of Aqp1 and Aqp3 remained unchanged, in the gills of *P. annectens* after 6 days of aestivation as compared with the control. It is apparent that changes in transcription preceded changes in translation and this can be viewed as a preparation for the aestivating lungfish to decrease the protein abundance of Aqp1 and Aqp3 in the subsequent maintenance phase of aestivation.

During the induction phase of aestivation, African lungfishes secrete large quantities of mucus while on land, and the mucus would dry up in 6–8 days to form a mucus cocoon which covers the complete body surface (Chew et al., 2004; Ip et al., 2005; Loong et al., 2005). There are evidences that mucus is produced and secreted by the skin (Sturla et al., 2001; see Chew et al., 2015 for a review), but the possibility of the gills contributing to mucus secretion, especially over the branchial surfaces, cannot be ignored. Sturla et al. (2001) reported that the gills of *P. annectens* were covered by a thick layer of mucus that filled the interlamellar spaces at the beginning of aestivation, presumably during the induction phase as the mucus cocoon was not fully formed. To date, the process of mucus secretion and the exact composition of the mucus of *P. annectens* are uncertain. Kesimer et al. (2010) has proposed a model of MUC5B mucin organization after granular release. Upon granular release, mucin granules undergo rapid unfolding into more linear structure due to the exchange of Ca^2+^ by Na^+^, which could stimulate the mucin gel to swell upon contact with ambient water (Kesimer et al., 2010). However, aestivation occurs on land and the gills of *P. annectens* are exposed to air and not water during the induction and maintenance phases. Therefore, it is possible that branchial water movements are needed to provide water to hydrate the mucus upon expansion and effectively spread the mucus over the gill surface. This could be facilitated by the unchanged protein abundance of Aqp1 and Aqp3 in the gills of *P. annectens* during the induction phase of aestivation as compared to those of the control fish. Although, trans-epithelial movement of water through the branchial epithelium is uncommon in fishes (Madsen et al., 2015), particularly among aquatic breathers, the gills of lungfishes may be atypical as they practice bimodal breathing in water and depend predominantly on pulmonary respiration on land.

Aqp3 is localized specifically to the basolateral membranes of ionocytes in the gills of several fishes (Deane and Woo, 2006; Tse et al., 2006; Giffard-Mena et al., 2007, 2008). By contrast, the localization of Aqp1 is more varied among different fish species. For *Coris julis* exposed to seawater, Aqp1 and Aqp3 are colocalized basolaterally with Na^+^/K^+^-ATPase in the chloride cells (Brunelli et al., 2010). Aqp1a is localized on the apical surface of the gill lamellae of seawater-acclimated *S. aurata* (Cerdà and Finn, 2010). The apical localization of Aqp1a in *S. aurata* is unexpected as significant water permeability of these cells might result in dehydration in a marine environment. However, it has been postulated that branchial Aqp1a could play other roles in transport of gases, such as CO_2_ or NH_3_ (Chen et al., 2010; Madsen et al., 2015). As the cell types in which Aqp1 and Aqp3 are expressed in the gills of *P. annectens* are uncertain at present, efforts should be made in the future to elucidate their cellular and subcellular localization which would provide insights into their probable functions in branchial cell volume regulation and/or transepithelial water movement.

### Decreases in transcript levels and protein abundance of *aqp1*/Aqp1 and *aqp3*/Aqp3 in the gills during the maintenance phase of aestivation

As it is essential to conserve metabolic fuels during long periods of aestivation without food intake, aestivation has often been associated with metabolic depression (Storey, 2002). Strong global suppression of gene expression and protein synthesis have been proposed as integral parts of metabolic rate depression because transcription and translation are energy-dependent processes (Storey and Storey, 2010). However, recent studies on *P. annectens* indicate that aestivation cannot be regarded as equivalent to a general depression of metabolism. Rather, aestivation involves structural and functional modifications of certain organs (Icardo et al., 2008, 2012; Ojeda et al., 2008) and the complex interplay between up-regulation and down-regulation of diverse cellular activities (see Ip and Chew, 2010; Chew et al., 2015 for reviews). Hiong et al. (2013) have reported that many more genes are up-regulated than down-regulated in the brain of *P. annectens* during the maintenance phase of aestivation, although one would expect exactly the opposite in a situation of metabolic depression. Ong et al. (2015) have also demonstrated that the gene and protein expression of *bhmt1*/Bhmt1 in the liver of *P. annectens* are up-regulated, with an increase in the protein expression of Bhmt in the muscle, after 6 months of aestivation. Similarly, Chng et al. (2014) have reported that 6 months of aestivation leads to significant increases in the mRNA expression of *argininosuccinate synthetase* and *argininosuccinate lyase* in the liver of *P. annectens*, which has to function, at least at a reduced rate, to catabolize amino acids and to detoxify ammonia to urea continuously during the maintenance phase of aestivation.

However, unlike the brain and liver, the gills of *P. annectens* apparently cease to function completely during the maintenance phase of aestivation, as they are covered with a thick layer of dried mucus with a reduction in the surface area of the branchial epithelium (Sturla et al., 2001). As expected, there were significant decreases in transcript levels and protein abundance of *aqp1*/Aqp1 and *aqp3*/Aqp3 in the gills of *P. annectens* after 6 months of aestivation. This can be interpreted as part of an overall mechanism to shut down branchial functions, which leads to a cessation of cell volume regulation in branchial epithelial cells. The suppression of transcription and translation of *aqp1*/Aqp1 and *aqp3*/Aqp3 would certainly reduce to a small extent the demand for metabolic energy. Furthermore, the decreases in protein abundance of Aqp1 and Aqp3 in the gills of *P. annectens* during the maintenance phase of aestivation might represent an additional strategy to reduce evaporative water loss through the branchial epithelium besides being covered with a thick layer of dried mucus.

Besides water, Aqp1/AQP1 can also facilitate CO_2_ conductance (Kaldenhoff et al., 2014). In *D. rerio*, which is an aquatic breather, branchial Aqp1 may play an alternative and fundamental role in CO_2_ (and NH_3_) transport (Chen et al., 2010). In water, CO_2_ excretion occurs mainly in the gills of lungfishes (Johansen and Lenfant, 1967; Lenfant and Johansen, 1968; Burggren and Johansen, 1986), except for *P. dolloi* which excretes CO_2_ predominantly through the lung (Perry et al., 2005). Hence, the possibility of branchial Aqp1 facilitating CO_2_ excretion in *P. annectens* immersed in water cannot be ignored. As the metabolic activity of *P. annectens* is reduced substantially during the maintenance phase of aestivation, it would logically lead to a decrease in CO_2_ production. However, branchial Aqp1 might not have a significant contribution to CO_2_ excretion when *P. annectens* is on land as the lung becomes the main respiratory organ (Laurent, 1996). Moreover, during aestivation, the gills are covered with a thick layer of dried mucus which would theoretically impede branchial gaseous exchange. Hence, the decreases in protein abundance of Aqp1 and Aqp3 in the gills of *P. annectens* during the maintenance phase of aestivation was probably unrelated to decreased CO_2_ production due to metabolic rate reduction.

### Partial recovery of Aqp1 and complete recovery of Aqp3 protein abundance in the gills during the arousal phase of aestivation

During the arousal phase of aestivation, it is essential for the lungfish to absorb water from the environment for rehydration, excrete urea accumulated during the maintenance phase and to regain the branchial functions. Riddle (1983) proposed that urea accumulated during the maintenance phase of aestivation could facilitate water uptake from the environment upon rehydration during arousal. However, water absorption must precede urea excretion because the urea accumulated in the body during the maintenance phase of aestivation is crucial to this osmotic phenomenon. Indeed, there was a three-fold increase in the transcript level of *aqp3* in the gills of *P. annectens* after 1 day of arousal from 6 months of aestivation, and by day 3, the protein abundance of Aqp3 returned to the control level. As Aqp3/AQP3 are known to support urea fluxes (Litman et al., 2009; Li and Wang, 2014), the complete recovery of Aqp3 protein abundance might facilitate urea excretion through the gills, apart from the skin (Hung et al., 2009), upon arousal. While the transcript level of *aqp1* in the gills of *P. annectens* after 1 day or 3 days of arousal remained significantly and substantially lower than that of the control, the protein abundance of Aqp1 recovered partially on day 3 of arousal. This indicates that for *aqp1*/Aqp1, translational regulation was more important than transcriptional regulation. Together, the increases in protein abundance of Aqp1 and Aqp3 denote the partial recovery of branchial function in relation to cell volume regulation and urea excretion.

### Perspectives

Results from this study reveal how *P. annectens* regulates *aqp1*/Aqp1 and *aqp3*/Aqp3 expression in the gills to cope with desiccation and rehydration during different phases of aestivation. As gills are also involved in iono-regulation and nitrogen waste excretion, future studies should extend the investigation to other branchial transporters/channels, such as Na^+^/K^+^-ATPase, urea transporter and Rhesus glycoproteins. Both the gill and the skin represent a direct interface between the interior and exterior environments. However, compared to the gill, there is a dearth of information on the expression and function of Aqp in the skin of fishes. Furthermore, the skin of *P. annectens* plays substantial roles in mucus secretion for cocoon formation during the induction phase of aestivation (Sturla et al., 2001; Chew et al., 2015), and in urea excretion during the arousal phase of aestivation (Hung et al., 2009). Hence, efforts should be made in the future to determine the effects of aestivation on the gene and protein expression of *aqp*/Aqp and other relevant transporters/channels in the skin of *P. annectens*.

## Note on abbreviations

Two different types of abbreviations were adopted in this report because the standard abbreviations of genes/proteins of fishes (http://wiki.zfin.org/display/general/ZFIN+Zebrafish+Nomenclature+Guidelines) are different from those of frogs and human/non-human primates (http://www.genenames.org). Specifically, for fishes, gene symbols are italicized, all in lower case, and protein designations are the same as the gene symbol, but not italicized with the first letter in upper case.

## Author contributions

YI designed the experiment. YC performed all experiments and analyzed the data. YI and YC wrote the manuscript. JO, BC, XC, KH, WW, and SC were involved in animal subjection and sample collection. SL and YI were involved in the analysis of data and approval of manuscript. All authors were involved in the revision of the manuscript.

### Conflict of interest statement

The authors declare that the research was conducted in the absence of any commercial or financial relationships that could be construed as a potential conflict of interest.
