# Supplementary material for: Molecular Characterization of Aquaporin 1 and Aquaporin 3 from the Gills of the African Lungfish, Protopterus annectens, and Changes in Their Branchial mRNA Expression Levels and Protein Abundance during Three Phases of Aestivation
Source: Front Physiol. 2016 Nov 10;7:532. doi: 10.3389/fphys.2016.00532 (PMC5102888; doi:10.3389/fphys.2016.00532)
Supplement: Supplementary file 1 [file Table1.DOCX]

# Supplementary tables

**Table S1. List of selected species and their accession numbers used for dendrogram analyses of Aqp1/AQP1.** “*” indicates the outgroup.

| Species | Accession number |
| --- | --- |
| *Acanthopagrus schlegelii* Aqp1 | ABO38816.1 |
| *Anabas testudineus* Aqp1aa | AGF30363.1 |
| *Anguilla anguilla* Aqp1a | CAD92027.1 |
| *Anguilla anguilla* Aqp1b | ABM26906.1 |
| *Anguilla japonica* Aqp1a | BAC82109.1 |
| *Anguilla japonica* Aqp1b | BAK53383.1 |
| *Bos taurus* AQP1 | ABF57368.1 |
| *Canis lupus familiaris* AQP1 | NP_001003130.1 |
| *Cynoglossus semilaevis* Aqp1 | ADG21868.1 |
| *Cyprinus carpio* Aqp1a1-1 | BAS18938.1 |
| *Danio rerio* Aqp1 | ACA29537.1 |
| *Danio rerio* Aqp1a | NP_996942.1 |
| *Dicentrarchus labrax* Aqp1 | ABI95464.2 |
| *Diplodus sargus* Aqp1 | AEU08496.1 |
| *Fundulus heteroclitus* Aqp1 | ACI49538.1 |
| *Homo sapiens* AQP1 | CAQ51480.2 |
| *Latimeria chalumnae* Aqp1 | XP_006005961.1 |
| *Mus musculus* AQP1 | EDK98728.1 |
| *Oryzias dancena* Aqp1 | BAN17349.1 |
| *Oryzias latipes* Aqp1 | XP_011485314.1 |
| *Osmerus mordax* Aqp1 | ACO09149.1 |
| *Ovis aries* AQP1 | AAB63463.1 |
| *Poecilia formosa* Aqp1 | XP_007548683.1 |
| *Pongo abelii* AQP1 | NP_001126220.1 |
| *Rattus norvegicus* AQP1 | EDL88090.1 |
| *Rhabdosargus sarba* Aqp1 | AEG78286.1 |
| *Rhinella marina* Aqp1 | AAA67782.1 |
| *Salmo salar* Aqp1b | NP_001133472.1 |
| *Sparus aurata* Aqp1a | ABM26907.1 |
| *Sparus aurata* Aqp1b | ABM26908.1 |
| *Sus scrofa* AQP1 | NP_999619.1 |
| *Takifugu obscurus* Aqp1 | ADG86337.1 |
| *Xenopus (Silurana) tropicalis* Aqp1 | NP_001005829.1 |
| *Xenopus laevis* Aqp1 | NP_001085391.1 |
| *Anopheles gambiae* Aqp1* | BAI60044.1 |
