# Supplementary material for: Molecular Characterization of Aquaporin 1 and Aquaporin 3 from the Gills of the African Lungfish, Protopterus annectens, and Changes in Their Branchial mRNA Expression Levels and Protein Abundance during Three Phases of Aestivation
Source: Front Physiol. 2016 Nov 10;7:532. doi: 10.3389/fphys.2016.00532 (PMC5102888; doi:10.3389/fphys.2016.00532)
Supplement: Supplementary file 2 [file Table2.DOCX]

# Supplementary tables

**Table S2. List of selected species and their accession numbers used for dendrogram analyses of Aqp3/AQP3.** “*” indicates the outgroup.

| Species | Accession number |
| --- | --- |
| *Anguilla anguilla* Aqp3 | CAC85286.1 |
| *Anguilla japonica* Aqp3 | BAH89253.1 |
| *Bos taurus* AQP3 | NP_001073262.1 |
| *Danio rerio* Aqp3a | AAH44188.1 |
| *Danio rerio* Aqp3b | NP_001159593.1 |
| *Dicentrarchus labrax* Aqp3 | ABG36519.1 |
| *Fundulus heteroclitus* Aqp3 | NP_001296892.1 |
| *Homo sapiens* AQP3 | AAY68214.1 |
| *Ictalurus punctatus* Aqp3 | AHH37605.1 |
| *Larimichthys crocea* Aqp3 | KKF15034.1 |
| *Latimeria chalumnae* Aqp3 | XP_006004793.1 |
| *Macaca mulatta* AQP3 | NP_001244972.1 |
| *Maylandia zebra* Aqp3 | XP_004544455.1 |
| *Mus musculus* AQP3 | BAB03270.1 |
| *Notothenia coriiceps* Aqp3 | XP_010776959.1 |
| *Oreochromis mossambicus* Aqp3 | BAD20708.1 |
| *Oreochromis niloticus* Aqp3 | AHY84681.1 |
| *Oryzias dancena* Aqp3 | BAP11294.1 |
| *Oryzias latipes* Aqp3 | XP_004072505.1 |
| *Pan troglodytes* AQP3 | JAA01153.1 |
| *Poecilia formosa* Aqp3 | XP_007562721.1 |
| *Poecilia reticulata* Aqp3 | XP_008417140.1 |
| *Rattus norvegicus* AQP3 | EDL98656.1 |
| *Rattus rattus* AQP3 | BAA04559.1 |
| *Sarotherodon melanotheron* Aqp3 | AHA92968.1 |
| *Sus scrofa* AQP3 | ABW06862.1 |
| *Takifugu obscurus* Aqp3 | ADG86338.1 |
| *Takifugu rubripes* Aqp3 | XP_003975282.1 |
| *Tribolodon hakonensis* Aqp3 | BAB83082.1 |
| *Xenopus (Silurana) tropicalis* Aqp3 | CAJ82459.1 |
| *Xenopus laevis* Aqp3 | NP_001081876.1 |
| *Ciona intestinalis* Aqp3* | XP_002131906.1 |
